# Deep-Learning-Based Detection of Infants with Autism Spectrum Disorder Using Auto-Encoder Feature Representation

**DOI:** 10.3390/s20236762

**Published:** 2020-11-26

**Authors:** Jung Hyuk Lee, Geon Woo Lee, Guiyoung Bong, Hee Jeong Yoo, Hong Kook Kim

**Affiliations:** 1 School of Electrical Engineering and Computer Science, Gwangju Institute of Science and Technology, Gwangju 61005, Korea; ljh0412@gist.ac.kr (J.H.L.); geonwoo0801@gist.ac.kr (G.W.L.); 2 Department of Psychiatry, Seoul National University Bundang Hospital, Seongnam-si, Gyeonggi-do 13620, Korea; 20409@snubh.org (G.B.); hjyoo@snu.ac.kr (H.J.Y.); 3 Department of Psychiatry, College of Medicine, Seoul National University, Seoul 03980, Korea

**Keywords:** auto-encoder, bidirectional long short-term memory (BLSTM), joint optimization, acoustic feature extraction, autism spectrum disorder

## Abstract

Autism spectrum disorder (ASD) is a developmental disorder with a life-span disability. While diagnostic instruments have been developed and qualified based on the accuracy of the discrimination of children with ASD from typical development (TD) children, the stability of such procedures can be disrupted by limitations pertaining to time expenses and the subjectivity of clinicians. Consequently, automated diagnostic methods have been developed for acquiring objective measures of autism, and in various fields of research, vocal characteristics have not only been reported as distinctive characteristics by clinicians, but have also shown promising performance in several studies utilizing deep learning models based on the automated discrimination of children with ASD from children with TD. However, difficulties still exist in terms of the characteristics of the data, the complexity of the analysis, and the lack of arranged data caused by the low accessibility for diagnosis and the need to secure anonymity. In order to address these issues, we introduce a pre-trained feature extraction auto-encoder model and a joint optimization scheme, which can achieve robustness for widely distributed and unrefined data using a deep-learning-based method for the detection of autism that utilizes various models. By adopting this auto-encoder-based feature extraction and joint optimization in the extended version of the Geneva minimalistic acoustic parameter set (eGeMAPS) speech feature data set, we acquire improved performance in the detection of ASD in infants compared to the raw data set.

## 1. Introduction

Autism spectrum disorder (ASD) is a developmental disorder with a high probability of causing difficulties in social interactions with other people [1]. According to the Diagnostic and Statistical Manual of Mental Disorders, Fifth Edition (DSM-5), ASD involves several characteristics such as being confined to specific interests or behaviors, delayed linguistic development, and poor functionality in terms of communicating or functioning in social situations [2]. As there is wide variation in terms of the types and severities of ASD based on its characteristics, the disorder is referred to as a spectrum [1]. Not only does ASD have the characteristics of a developmental disorder with a life-span disability, but its prevalence is also increasing—from 1 in 150 children in 2000 to 1 in 54 children in 2016 [3]. As diverse evidence has been obtained from previous research showing that the chance of improvement in the social abilities of people with ASD increases when an earlier clinical intervention is performed [4], the early detection of ASD characteristics has become a key point of current ASD research.

Various instruments for discriminating ASD have been developed, and the commonly accepted gold standard schemes are behavioral assessments, which are time-consuming procedures and require multidisciplinary teams (MDTs). However, most behavioral assessments suffer in terms of the stability of their ASD diagnosis as a result of the issues of accessibility or subjectivity and interpretive bias between professions [5]. Therefore, several attempts to develop objective and precise diagnostic methods have been made in multiple fields, such as genetic determination [6], principle analysis of brain images [7], and physiological approaches [8].

One prominent area of behavioral observations is that of infants’ vocal characteristics. Children with ASD are known to have abnormalities in their prosody resulting from deficits in their ability to recognize the inherent mental conditions of others [9], and their atypical vocalizations are known to be monotonous or exaggerated, which can be revealed using various acoustic characteristics, followed by engineering approaches for the discrimination of ASD or typical development (TD) in children based on the vocal and acoustic features. For example, in [10], the researchers estimated deficits in the vocalization of children with ASD at an average age of 18 months, such as “flat” intonation, atypical pitch, or control of volume based on the variability of pitch and the long-term average spectrum (LTAS) using fast Fourier transform, where significant differences were observed in the spectral components at low-band frequencies, as well as spectral peaks and larger pitch ranges and standard deviations. The development of linguistic abilities is also considered to be a distinguishable feature of delayed development in children with ASD. Earlier vocal patterns at age 6–18 months were proven to be differentiable in a study [11] that aimed to confirm the hypothetical vocal patterns and social quality of vocal behavior in order to differentiate between ASD and TD cohorts in groups of children aged 0–6, 6–12, and 12–18 months in terms of categorized speech patterns consisting of vocalization, long reduplicated babbling, two-syllable babbling, and first words. Evidence of abnormalities in children with ASD were shown, in these cases, as a significant decrease in vocalization and first word rate, while the difference in babbling ability between children with ASD and TD was negligible.

Given the development and improvement of machine learning algorithms, as the achievement in the performance of state-of-the-art classification and discrimination tasks [12], recent attempts to develop automated classification methods based on machine learning techniques have been based on the distinctiveness of vocal characteristics, and have been shown to be promising alternatives to the conventional methods in many publications [13]. For examples of machine learning classification, the researchers of [14] employed various acoustic–prosodic features, including fundamental frequency, formant frequencies, harmonics, and root mean square signal energy. In their research, support vector machines (SVMs) and probabilistic neural networks (PNNs) were adopted as classifiers, which showed effectual accuracy in discriminating children with ASD from children with TD. Meanwhile, the authors of [15] employed more recent deep learning techniques, such as convolutional neural networks (CNNs) and recurrent neural networks (RNNs) with spectral features from short-time Fourier transform (STFT) and constant Q transform (CQT), to classify children diagnosed using the autism diagnostic observation schedule (ADOS), also showing promising results in multiple outcomes from SVMs, RNNs, and a combination of CNN and RNN classifiers.

A generalized acoustic feature set, an extended version of the Geneva minimalistic acoustic parameter set (eGeMAPS) [16], and the bidirectional long short-term memory (BLSTM) model were adopted to differentiate between children with ASD and children with TD in [17], showing that 75% of the subjects’ utterances were correctly classified with the simple application of a deep learning model and feature sets. While the quality of previous research based on various acoustic features has proven the effectiveness of acoustic features and classification algorithms for the detection of abnormalities in children’s voices in ASD group compared to those of TD group, the complexity and relationship being inherent between the features will remain uncertain until a large amount of data can be accumulated. Furthermore, a limitation still remains in terms of the problems regarding data collection, since there are difficulties pertaining to the need to secure the anonymity of infant subjects, as well as the unintended ignorance of parents at earlier stages of their infant’s development. The data of infants are, accordingly, dispersed by gender, age, and number of vocalizations, or consist of comparably small volumes of audio engineering data in general. These problems were typically overlooked by previous research with controlled and small amounts of data.

In order to provide suggestions for a method to overcome the abovementioned restrictions, we focus on examining the feasibility of neural networks as a feature extractor, employing an auto-encoder (AE), which can modify acoustic features into lowered and separable feature dimensions [18]. We construct a simple six-layered stacked AE that contains an input layer, three fully connected (FC) layers, an output layer, and one auxiliary output layer, which has categorical targets for ASD and TD for the optimization of the latent feature space of the AE. We train the AE and deep learning models and compare the results for each model based on SVMs and vanilla BLSTM, while adopting the same model parameters from the method suggested in [17].

The remainder of this paper is organized as follows. Section 2 describes the specifications of the participants’ data, data processing, feature extraction, statistical analysis, and experimental setup. Section 3 presents the performance evaluations for each algorithm of the SVMs and vanilla BLSTM. Lastly, Section 4 concludes the paper.

## 2. Proposed Method

### 2.1. Data Collection and Acoustic Feature Extraction

This study was based on the audio data from video recordings of ASD diagnoses, which were collected from 2016 to 2018 at Seoul National University Bundang Hospital (SNUBH). We received approval from the Institutional Review Board (IRB) at SNUBH to use fully anonymized data for retrospective analysis (IRB no: B-1909/567-110) from existing research (IRB no: B-1607/353-005). We collected the audio data of 39 infants who were assessed using seven multiple instruments, consisting of (1) ADOS, second edition (ADOS-2), (2) the autism diagnostic interview, revised (ADI-R), (3) the behavior development screening for toddlers interview (BeDevel-I), (4) the behavior development screening for toddlers play (BeDevel-P), (5) the Korean version of the childhood autism rating scale (K-CARS) refined from CARS-2, (6) the social communication questionnaire (SCQ), and (7) the social responsiveness scale (SRS) [19,20,21,22]. The final diagnosis was based on the best clinical estimate diagnosis according to the DSM-5 ASD criteria by a licensed child psychiatrist using all of the available participant information. The participants’ ages ranged between 6 and 24 months, where the average age was 19.20 months with a standard deviation (SD) of 2.52 months. Note here that the age means the age at the time when each infant visited the hospital to undergo an initial diagnosis examination. There were four males and six females diagnosed with ASD, whose average age was 14.72 months with a SD of 2.45. The remaining participants consisted of TD children (19 males and 10 females). Table 1 displays the collected data distribution, while Table 2 shows detailed information of collected data from the infants.

As each infant’s audio data were recorded during the clinical procedure to elicit behaviors from infants, with the attendance of one doctor or clinician and one or both parents with the child in the clinical area, the audio components consisted of various speeches from the child, the clinician, and the parent(s), as well as noises from toys or dragging chairs. Note here that the recordings were done in one of two typical clinical rooms in SNUBH, where the room dimensions were 365 cm × 400 cm × 270 cm and 350 cm × 350 cm × 270 cm, and the hospital noise level was around 40 dB. In order to analyze the vocal characteristics of the infants, each audio clip was processed and split into audio segments containing the infant’s voice, not disturbed by music or clattering noises from toys or overlapped by the voices of the clinician or parent(s). Each segment was classified into one of five categories, labeled from 0 to 4, for measuring the data distribution. Each label was intended to show differentiable characteristics relative to the children’s linguistic development: (1) 0 for one syllable, which is a short, momentary single vocalization such as “ah” or “ba”; (2) 1 for two syllables, commonly denoted as canonical babbling, as a reduplication of clear babbling of two identical or variant syllables such as “baba” or “baga”; (3) 2 for babbling, not containing syllables; (4) 3 for first word, such as “mother” or “father”; and (5) 4 for atypical voice, including screaming or crying. The distribution of each type of vocalization in seconds is shown in Table 3. The number of vocalizations per category is presented along with a rational value considering the difference between the ASD and TD groups. While the data were unbalanced and very small, the distribution of ASD and TD vocalizations show the same tendency as reported in [10], where the ASD group showed a significantly lower ratio of first words and an increased ratio of atypical vocalizations, revealing developmental delay in linguistic ability.

For acquiring qualified and effective feature sets for the vocal data, eGeMAPS was employed for voice feature extraction. GeMAPS is a popular feature set providing minimalistic speech features generally utilized for automatic voice analysis rather than as a large brute force parameter set. As an extended version, eGeMAPS contains 88 acoustic features that were fully utilized in this experiment. Each recorded set of audio data stored as a 48 kHz stereo file was down-sampled and down-mixed into a 16 kHz mono-audio file, taking into consideration its usability and resolution in mel-frequency cepstral coefficients (MFCCs). To extract the speech features for ASD classification, each infant’s utterances were segmented into 25 ms frames with a 10 ms overlap between frames. Then, 88 different features of the eGeMAPS were extracted for each frame with open source speech and music interpretation using the large-space extraction (OpenSMILE) toolkit [23], and these features were normalized by mean and standard deviation. The normalization scaling was acquired and fixed by normalizing the factors of the training data set. The features were grouped for each five frames considering the time-relevant characteristics of the speech data.

### 2.2. Pre-Trained AE for Acoustic Features

To further process and refine the acoustic data, a feature-extracting AE was introduced. An AE is a hierarchical structure that is trained as a regression model for reproducing the input parameters. The AE takes inputs and converts them into latent representations, and then reconstructs the input parameters from the latent values [24]. If we consider an input of AE, $x\in R^{d}$, then the latent representation $z\in R^{d^{\prime }}$ and the reconstruction of the input $y\in R^{d}$ are obtained by applying a nonlinear activation function f to the weight sum of z using a weighting matrix $W\in R^{d\times d^{\prime }}$ and a bias vector $b\in R^{d^{\prime }}$, such as

(1) $$z=f\left(W^{T}x+b\right)$$

(2) $$y=f\left(W^{T}z+b^{\prime }\right)$$

where *T* is a matrix transpose operator. When the latent dimension $d^{\prime }<d$, the output from the latent layer is considered to be a compressed, meaningful value extracted from the input, which is also noted as a bottleneck feature [25].

The normalized eGeMAPS features were applied to train the feature-extracting AE, applying the same data as the input and the target. The AE model contained a latent layer with a lowered, compacted feature dimension compared to the input layer to achieve the useful bottleneck feature. The model was symmetrically structured, centering around the latent layer, and the model could be divided into two components: the encoder, consisting of layers from the input to the latent layers, and a decoder, consisting of layers from the bottleneck to the output layers.

**Figure 1:**
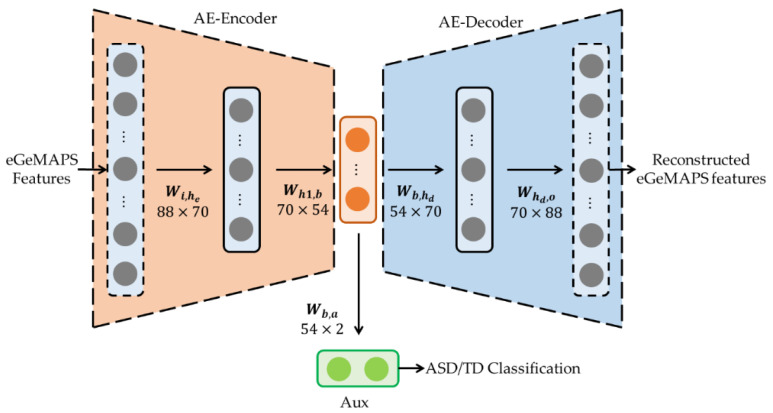
Structure of a semi-supervised auto-encoder (AE) model. eGeMAPS, extended version of the Geneva minimalistic acoustic parameter set; ASD, autism spectrum disorder; TD, typical development.

The AE structure is depicted in Figure 1. Our AE model consisted of FC layers, with the dimensions of 88, 70, 54, 70, and 88 nodes for the input, hidden, latent, hidden, and output layers, respectively. The hidden dimension was selected experimentally and the bottleneck feature dimension was used for comparison with previous research [17], where 54 features were selected considering the statistical dissimilarity of the distributions between the ASD and TD features based on the Mann–Whitney *U* test [26]. We additionally introduced an auxiliary output as the binary categorical target for ASD and TD, which is known as the semi-supervised method, to train the AE model effectively [27]. The auxiliary output is depicted as Aux in Figure 1. The reconstructed features and auxiliary classification can be written as

(3) $$z_{i}=f\left(W_{i-1,i}z_{i-1}+b_{i-1,i}\right)$$

where $z_{1}=f\left(W_{0,1}x+b_{0,1}\right)$, and

(4) $$y_{rec}=W_{3,4}z_{3}+b_{3,4}$$

(5) $$\;\;y_{aux}=\partial \left(W_{2,a}z_{2}+b_{2,a}\right)$$

where $y_{rec}$ refers to the reconstructed eGeMAPS features, $y_{aux}$ is the auxiliary classification result, f is the activation function, and ∂ is the softmax activation.

The losses of the reconstruction error for main AE target are measured using the mean absolute error, while the auxiliary ASD/TD target loss is the binary cross-entropy, and they are added and simultaneously optimized with rational hyper-parameters. The overall loss equation is

(6) $$L_{recon}=\frac{1}{N}\overset{N}{\underset{i=1}{\sum }}\left|y_{i_{rec}}-y_{i_{gt}}\right|$$

(7) $$L_{aux}=-y_{1_{gt}}\log\left(y_{1_{aux}}\right)-\left(1-t\right)y_{1_{gt}}\log\left(y_{1_{aux}}\right)$$

(8) $$L_{total}=L_{recon}+\alpha L_{aux}$$

where $L_{recon}$, $L_{aux}$, and $L_{total}$ denote the reconstruction error, auxiliary loss using a binary cross-entropy loss function, and total loss, respectively.

For our stacked AE model, a rational value of α=0.3 was selected experimentally, considering the proportion of each loss. In order to train the AE effectively, both L2 normalization for weight normalization and batch normalization were adopted [28,29]. After the training was completed, we fetched the encoder of the AE as the feature extraction part for the joint optimization model in the training procedures of the deep learning model.

### 2.3. Establishing and Training the Deep Learning Model for ASD Detection

**Figure 2:**
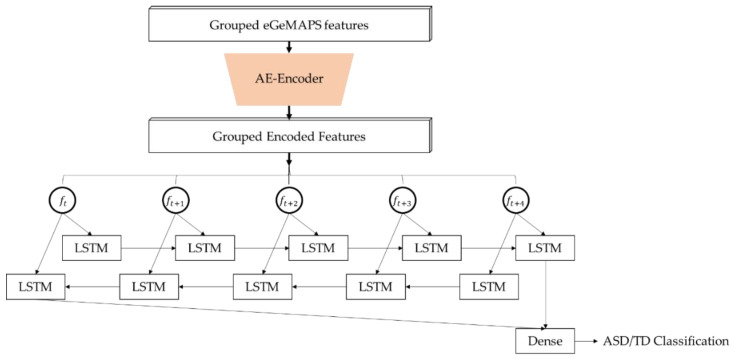
Structure of a joint optimization model of an auto-encoder (AE) and bidirectional long short-term memory (BLSTM).

As the eGeMAPS data were set and the AE was trained through semi-supervised learning, the machine learning models, such as SVMs, BLSTM, and joint optimized BLSTM were constructed. Each model had its own input parameter dimensions and the same output targets as ASD and TD classification labels. The eGeMAPS feature data were paired with the diagnostic results for the supervised learning of the neural network models. For the binary decision, ASD was labeled as a positive data point, with a label of (0, 1), while TD was labeled as a negative data point (1, 0). We composed four kinds of models with the paired data: SVMs with linear kernel, the vanilla BLSTM with 88 eGeMAPS features, the vanilla BLSTM with 54 eGeMAPS features, and the jointly optimized BLSTM layer with the AE. The joint optimization model is depicted in Figure 2. As the data set was prepared as the input with five sequential frames, i.e., the grouped eGeMAPS features in Figure 2, the SVMs received a single frame parameter of 440 dimension which was flattened from the original five input frames. For the deep learning models, batch normalization, rectangular linear unit (ReLU) activation, and dropout were applied for each layer, except for the output layer [30,31], and the adaptive momentum (ADAM) optimizer [32] was used to train the network. The training procedure was controlled by early stopping for minimizing the validation error with 100 epoch patience, while saving the best models for improvement of the validation loss by each epoch. Because the amount of speech data was relatively small for a deep learning model compared to the disparate field of audio engineering, we grouped the data into five segments, while the test utterances were separated formerly, which were selected randomly for 10% of the total data, were evenly distributed across each vocalization type, and underwent five-fold cross-validation for training; then, the best-performing model was chosen. Our model was trained with the TensorFlow framework [33]. For comparison, an SVM model with linear kernel was trained with the same data split as the proposed deep learning model, and as well as the vanilla BLSTM suggested in [17], which has single BLSTM with eight cells.

## 3. Performance Evaluation

The performance of each method was evaluated through five-fold cross validation, where 95 average ASD utterances and 130 average TD utterances were proportionally distributed over five cases of vocalizations for the generalized estimation of unconcentrated utterance data. The averaged performances of the five validation splits of each model are described in Table 4. The labeled names of the BLSTM were used as the features for training the BLSTM model, where eGeMAPS-88 denotes 88 features of eGeMAPS, eGeMAPS-54 denotes 54 features selected by the Mann–Whitney *U* test, and AE-encoded denotes the joint optimized model. In the classification stage, one utterance was processed in the frame-wise method and the softmax output was converted to class indices 0 and 1, and if the average of class indices of the frames was over 0.5, then the utterance was considered an ASD child’s utterance. The performances were scored with conventional measures, as well as unweighted average recall (UAR) and weighted average recall (WAR), chosen in the INTERSPEECH 2009 Emotion challenge, which considered imbalanced classes [34]. In the experiment, the SVM model showed very low precision, which was extremely biased toward the TD class. The BLSTM classifier with 88 features of eGeMAPS and the AE model showed considerable quality in terms of classifying ASD and TD children, while the AE model showed only marginal improvement in correctly classifying children with ASD compared to eGeMAPS-88. The 54 selected features showed degraded quality compared to eGeMAPS-88, obtaining more biased results toward children with TD.

## 4. Discussion

The vanilla BLSTM model presented in [17] conducted discrimination on well-classified subjects with 10-month-old children and sorted 54 features from eGeMAPS that had a distinctive distribution between ASD and TD selected by the Mann–Whitney *U* test using the three-fold cross-validation method. However, because the difference in the data distribution failed to achieve the same eGeMAPS feature selection between the test and classification results with the specified feature set presented herein, the application of an identical model structure and the adoption of the same feature domain will allow both approaches to be indirectly comparable.

**Figure 3:**
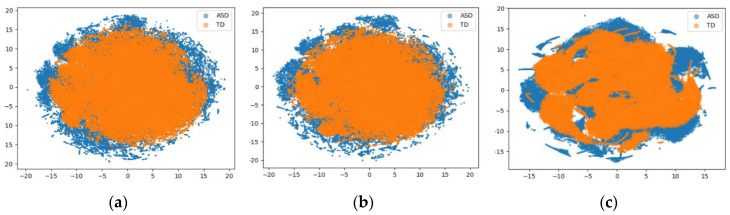
Two-dimensional scatter plot for (**a**) eGeMAPS-88, (**b**) eGeMAPS-54, and (**c**) the AE processed by t-stochastic neighbor embedding (t-SNE).

These results can be interpreted by the data distributions, and we performed t-stochastic neighbor embedding (t-SNE) analysis [35] on the training data set, which can nonlinearly squeeze the data dimension based on a machine learning algorithm. Figure 3 shows each data distribution as a two-dimensional scatter plot. In the figure, the eGeMAPS features from eGeMAPS-88 and eGeMAPS-54 showed almost identical distribution, except for the amount of ASD outliers, which implies that the ASD and TD features in the eGeMAPS features show similar distributions in this experiment. As shown in [16], eGeMAPS includes temporal features that are relevant to vocalizations and utterances; thus, these features might cause confusion regarding the discrimination between ASD and TD. The AE-encoded features, however, showed a redistributed feature map with a more characteristic distribution compared to the eGeMAPS features. This is because the AE-encoded features were compressed into a bottleneck feature, which was derived by weighting the matrix, paying attention to the significant parameters while reducing the influence from the ambiguous parameters. While the joint optimization model achieved only marginally improved results compared to eGeMAPS-88, the distribution of the feature map would be more noticeable in improved feature extraction models, as well as more differentiable in complex models, although BLSTM with eight cells was employed for a comparison with conventional research in this experiment.

While the overall performance scores were comparably low for general classification problems on account of the subjectivity and complexity of problems, and the limitation in terms of the shortage of data, the results of the jointly optimized model imply the possibility of deep-learning-based feature extraction for the improvement of automated ASD/TD diagnosis under restricted circumstances.

## 5. Conclusions

In this paper, we conducted experiments for discovering the possibility of auto-encoder-based feature extraction and a joint optimization method for the automated detection of atypicality in voices of children with ASD during early developmental stages. Under the condition of an insufficient and dispersed data set, the classification results were relatively poor in comparison to the general classification tasks based on deep learning. Although our investigation used a limited number of subjects and an unbalanced data set, the suggested auto-encoder-based feature extraction and joint optimization method revealed the possibility of feature dimension and a slight improvement in model-based diagnosis under such uncertain circumstances.

In future work, we will focus on increasing the reliability of the proposed method by addition of a number of infants’ speech data, refinement of the acoustic features, an auto-encoder for feature extraction, and better, deeper, and up-to-date model structures. This research can also be extended to children with the age of 3 or 4 who can speak several sentences. In this case, we will investigate the linguistic features, as well as acoustic features, such as we have done in this paper. In addition to ASD detection, this research can be applied to the detection of infants with development delays.

## Figures and Tables

**Table 1:** Distribution of age and gender (male/female).

Ages (Month)	No. of Subjects Diagnosed as ASD	No. of Subjects Diagnosed as TD	No. of Infant Subjects
6–12 months	0	5 M/1 F	5 M/1 F
12–18 months	1 M/3 F	14 M/9 F	15 M/12 F
18–24 months	3 M/3 F	0	3 M/3 F
Age (average ± SD)	19.20 ± 2.52	14.72 ± 2.45	15.92 ± 3.17

**Table 2:** Detailed information on the age, gender, and initial and definite diagnosis dates of each infant in Table 1.

Infant ID	Age (Months) on Initial Diagnosis Date	Gender	Initial Diagnosis Date (Year/Month/Day)	Definite Final Diagnosis Date (Year/Month/Day)	ASD/TD
1	18	Male	2018/07/28	2018/08/28	TD
2	18	Male	2017/07/27	2017/08/27	TD
3	10	Male	2018/08/10	2018/09/10	TD
4	13	Male	2017/06/10	2017/07/10	TD
5	22	Female	2018/01/31	2018/02/28	ASD
6	16	Male	2018/03/17	2018/04/17	TD
7	17	Female	2018/06/30	2018/07/30	TD
8	14	Female	2018/01/06	2018/02/06	TD
9	18	Male	2018/07/17	2018/08/17	TD
10	14	Male	2017/11/04	2017/12/04	TD
11	17	Female	2017/06/29	2017/07/29	ASD
12	12	Female	2018/01/20	2018/02/20	TD
13	9	Male	2017/02/18	2017/03/18	TD
14	18	Female	2017/03/04	2017/04/04	ASD
15	18	Male	2018/05/19	2018/06/19	TD
16	24	Female	2018/08/08	2018/09/08	ASD
17	19	Male	2018/02/24	2018/03/24	ASD
18	19	Male	2017/04/18	2017/05/18	ASD
19	18	Female	2017/03/04	2017/04/04	TD
20	12	Male	2016/12/31	2017/01/31	TD
21	16	Female	2018/03/16	2018/04/16	TD
22	20	Male	2017/10/14	2017/11/14	ASD
23	15	Male	2018/05/09	2018/06/09	ASD
24	17	Female	2017/02/04	2017/03/04	TD
25	16	Male	2018/03/17	2018/04/17	TD
26	12	Male	2018/03/29	2018/04/29	TD
27	17	Female	2017/01/25	2017/02/25	TD
28	17	Male	2018/02/08	2018/03/08	ASD
29	14	Male	2018/01/13	2018/02/13	TD
30	16	Male	2016/11/30	2016/12/30	TD
31	12	Male	2017/03/22	2017/04/22	TD
32	15	Male	2017/03/11	2017/04/11	TD
33	16	Male	2017/12/05	2018/01/05	TD
34	13	Female	2017/12/13	2018/01/13	TD
35	15	Female	2017/03/25	2018/04/25	TD
36	13	Male	2018/08/25	2018/09/25	TD
37	21	Male	2017/06/24	2017/07/24	ASD
38	14	Male	2017/02/22	2017/03/22	TD
39	14	Male	2018/01/27	2018/02/27	TD

**Table 3:** Amount (ratio) of each type of vocalization in seconds.

Vocal Label	ASD	TD
0	80.134 (0.104)	267.897 (0.250)
1	314.405 (0.409)	443.498 (0.414)
2	33.241 (0.043)	34.766 (0.032)
3	8.311 (0.011)	57.286 (0.054)
4	333.400 (0.433)	266.794 (0.249)
Total	769.491	1070.241

**Table 4:** Classification results from the support vector machine (SVM), BLSTM with 88 or 54 eGeMAPS features, 54 selected eGeMAPS features, and BLSTM with AE-encoded features.

Models	SVM		BLSTM (eGeMAPS-54)		BLSTM (eGeMAPS-88)		BLSTM (AE-Encoded)	
Predicted To	ASD	TD	ASD	TD	ASD	TD	ASD	TD
ASD	62	18	170	103	196	99	215	98
TD	413	632	305	547	279	551	260	552
Accuracy	0.6178		0.6373		0.6640		0.6818	
Precision	0.1305		0.3579		0.4126		0.4526	
Recall	0.7750		0.6227		0.6644		0.6869	
F1 score	0.2234		0.4545		0.5091		0.5457	
UAR	0.5514		0.5997		0.6302		0.6509	

UAR, unweighted average recall.
